# Combined Posterolateral Corner and Proximal Tibiofibular Joint Anatomic Reconstruction: A Surgical Strategy for Complex Posterolateral Knee Instability

**DOI:** 10.1111/os.70394

**Published:** 2026-08-12

**Authors:** Javier Faus‐Cotino, Isabel Andia, Aingeru Sarriugarte Lasarte, Ainhoa Amarilla‐Irusta, José Antonio Guerrero Serrano

**Affiliations:** ^1^ Department of Traumatology and Orthopedic Surgery Cruces University Hospital Barakaldo Spain; ^2^ Regenerative Therapies Biocruces Bizkaia Health Research Institute, Cruces University Hospital Barakaldo Spain; ^3^ Department of General Surgery, Cruces University Hospital Cruces University Hospital Barakaldo Spain; ^4^ Department of Surgery UPV/EHU Leioa Spain; ^5^ Innovation in Surgery, Transplantation, and Health Technologies Biocruces Bizkaia Health Research Institute, Cruces University Hospital Barakaldo Spain; ^6^ Immunopathology Group Biocruces Bizkaia Health Research Institute, Cruces University Hospital Barakaldo Spain; ^7^ Department of Traumatology and Orthopedic Surgery 12 de Octubre Universitary Hospital Madrid Spain

**Keywords:** anatomic reconstruction, knee instability, posterolateral corner; proximal tibiofibular joint

## Abstract

**Objective:**

Concomitant injury of the posterolateral corner (PLC) and the proximal tibiofibular joint (PTFJ) is a complex clinical entity that currently lacks a standardized surgical treatment. Failure to address PTFJ instability can compromise the result of PLC reconstructions. Therefore, the purpose of this study is to describe a surgical technique for simultaneous stabilization of the PLC and the PTFJ in the setting of a combined injury.

**Methods:**

An anatomic ligament reconstruction of the PLC and PTFJ was performed on a cadaveric knee specimen using two tendon autografts. Tunnel placement and graft passage were guided by anatomical landmarks. A sequential fixation strategy was implemented, allowing independent graft tensioning and fixation at predetermined knee flexion angles. Qualitative knee stability was assessed comparatively before and after reconstruction using standard examination maneuvers.

**Results:**

The described technique allowed an anatomic reconstruction of both the PLC and the PTFJ. Reconstruction of the proximal tibiofibular ligaments provided a stable structural base at the fibular head, enhancing the reliability of the PLC reconstruction. Independent graft fixation enabled controlled tensioning consistent with known biomechanical principles.

**Conclusions:**

This technical note describes an anatomic technique for combined PLC and PTFJ stabilization in a complex injury pattern for which no standardized surgical solution has been defined in the literature. This technique may serve as a foundation for future biomechanical and clinical studies.

## Graphical abstract

A novel anatomic surgical strategy for simultaneous stabilization of the posterolateral corner and proximal tibiofibular joint is described. By using independent graft tensioning and angle‐specific fixation, this reproducible technique successfully restores global knee stability, addressing a complex injury pattern previously lacking a standardized treatment.

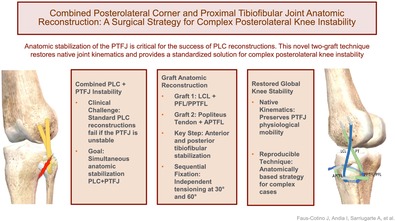

## 1 Introduction

The concomitant injury of the posterolateral corner (PLC) and the proximal tibiofibular joint (PTFJ) represents a complex, frequently underrecognized, and undertreated clinical entity. Although often overlooked, PTFJ instability is relatively prevalent within the spectrum of multiligamentous knee injuries, as current literature indicates that the PTFJ is injured in up to 9% of such cases, particularly those involving the PLC [1, 2].

**FIGURE 1:**
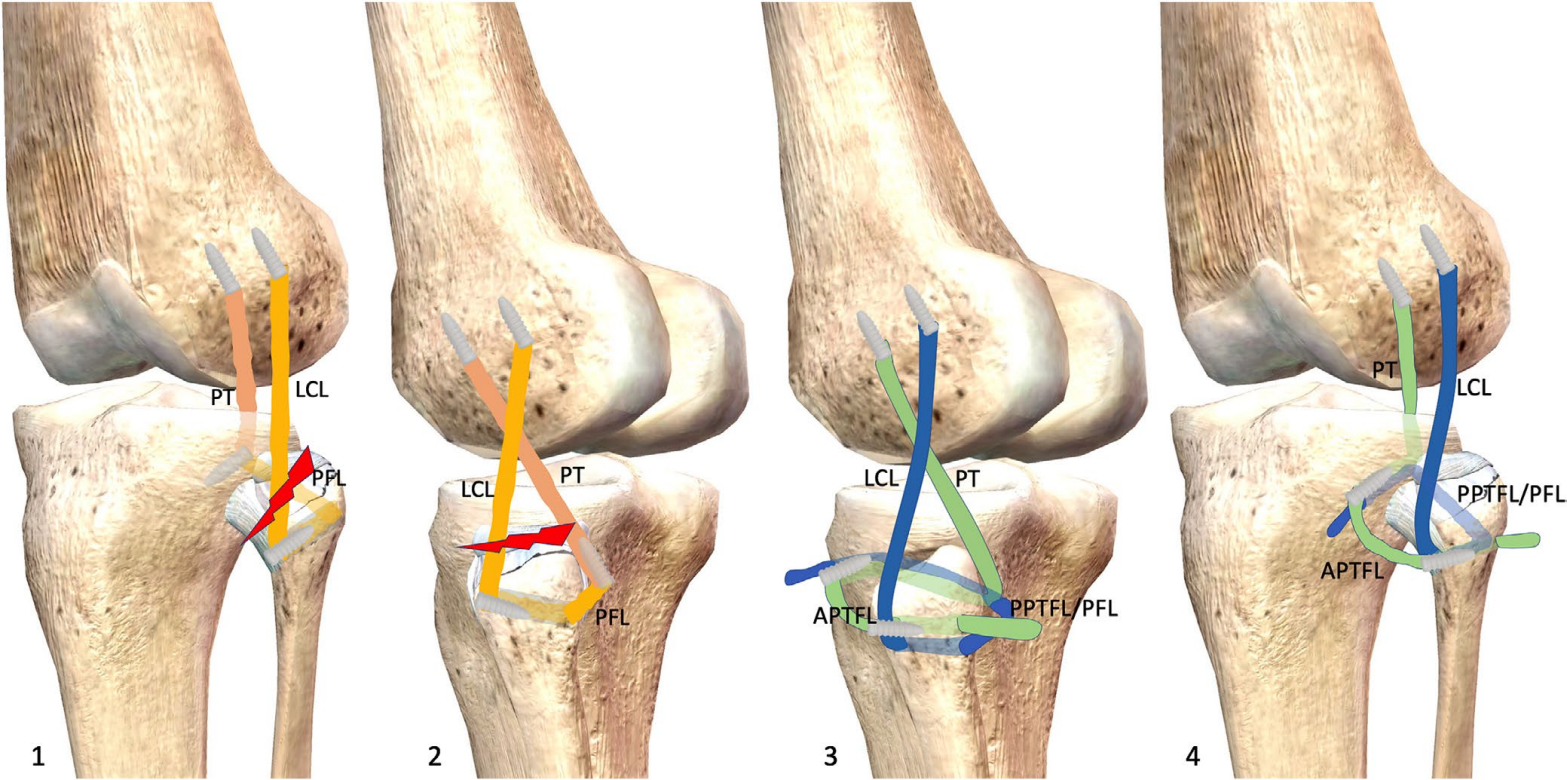
Failure of isolated PLC reconstruction in the presence of proximal tibiofibular joint instability. Knees 1 and 2: In the presence of a proximal tibiofibular joint injury (represented as a red bolt icon), isolated anatomical reconstruction of the PLC fails to yield a stable construct (the example illustrates a LaPrade‐type reconstruction). Knees 3 and 4: The proposed surgical alternative incorporates an additional graft passage between the tibia and fibula anteriorly, aimed at stabilizing the PTFJ. APTFL, anterior proximal tibiofibular ligament; LCL, lateral collateral ligament; PFL, popliteofibular ligament; PPTFL, posterior proximal tibiofibular ligament; PT, popliteus tendon.

The PLC, comprising the lateral collateral ligament (LCL), popliteus tendon (PT), and popliteofibular ligament (PFL), acts as a primary restraint to varus and external rotation forces, remaining essential for global knee stability [2, 3, 4, 5]. Several key stabilizing structures of the PLC insert on the fibular head; consequently, the integrity of the PTFJ is critical both in the setting of PLC injury and for the success of PLC reconstruction techniques. Unaddressed instability of the PTFJ compromises the effectiveness and durability of PLC reconstruction if not addressed concurrently. Furthermore, current evidence indicates that standard anatomical PLC reconstructions, such as those described by LaPrade or Arciero, fail to provide adequate stability if concomitant instability of the PTFJ is present (Figure 1). Therefore, rigorous evaluation of the PTFJ is critical in patients with PLC injuries, and targeted treatment should be performed when instability is detected [2, 6].

Despite the recognized biomechanical interdependence between the PLC and the PTFJ, a standardized surgical strategy for the simultaneous reconstruction of both entities has yet to be established. While an increasing frequency of published reports has documented numerous clinical cases involving this concomitant injury pattern, current surgical management remains characterized by a heterogeneous array of techniques lacking a definitive gold‐standard approach [2]. Current approaches to the combined injury often rely on isolated PLC reconstruction, arthrodesis of the PTFJ, or the expectation of spontaneous healing of the PTFJ capsule and ligaments. However, the intrinsic healing potential of the PTFJ remains poorly documented, and extrapolated evidence from other synovial joints suggests limited capacity for spontaneous healing of intra‐articular ligamentous structures due to the inhibitory effects of synovial fluid [2, 7, 8, 9, 10, 11, 12, 13, 14, 15, 16, 17, 18, 19, 20]. This biological limitation implies that achieving lasting PTFJ stability likely requires active surgical intervention. Accordingly, anatomic ligament reconstruction represents a biologically and biomechanically sound alternative to arthrodesis, as it preserves native joint kinematics while restoring stability [8, 9, 10, 20].

The purposes of this study are: (i) to describe and technically validate an anatomic reconstruction technique that simultaneously restores stability to both the PLC and the PTFJ by reconstructing the LCL, PT, and PFL, in addition to the posterior and anterior proximal tibiofibular ligaments; and (ii) to describe a sequential fixation strategy that allows independent tensioning at specific knee flexion angles.

## 2 Methods

### 2.1 Rationale and Concept of the Combined PLC–PTFJ Reconstruction

**FIGURE 2:**
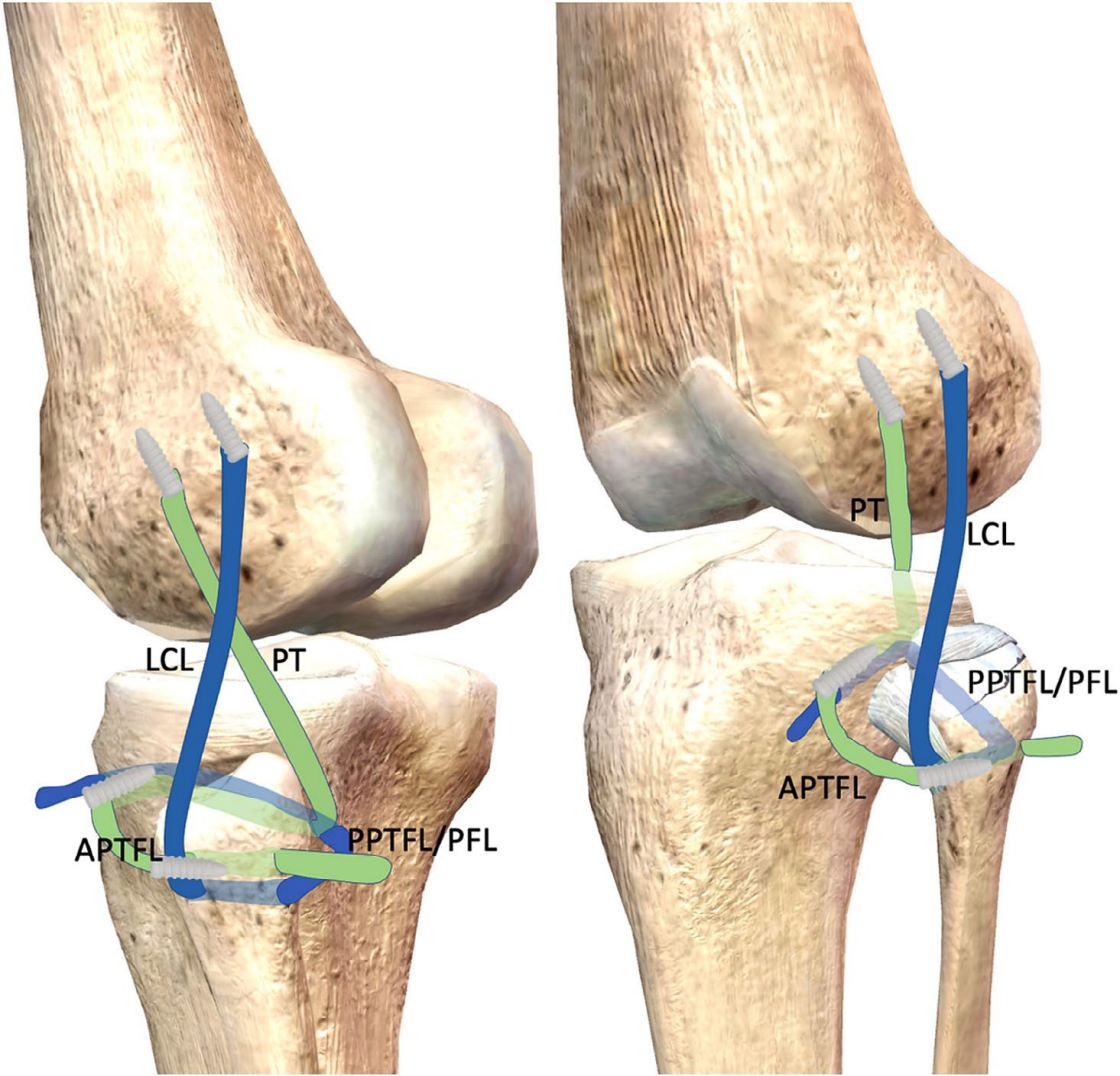
To achieve stabilization of the PLC and the PTFJ through combined ligamentous reconstruction, an anterior graft passage between the tibia and fibula is required. The figure represents the performed configuration in the cadaveric model utilizing two grafts, one reconstructing the LCL and the popliteofibular ligament/posterior proximal tibiofibular ligament, while the second reconstructs the popliteus tendon and the anterior proximal tibiofibular ligament. APTFL, anterior proximal tibiofibular ligament; LCL, lateral collateral ligament; PFL, popliteofibular ligament; PPTFL, posterior proximal tibiofibular ligament; PT, popliteus tendon.

Isolated anatomic reconstruction of the PLC may fail in the presence of PTFJ instability, as persistent fibular instability can compromise graft function and overall construct integrity. In the presence of a PTFJ injury, isolated anatomical reconstruction of the PLC fails to yield a stable construct because the PTFJ remains unstable (the only intact structure limiting the mobility of the fibula relative to the tibia is the reconstructed popliteofibular ligament, as the proximal tibiofibular ligaments are injured), thereby compromising the global integrity of the reconstruction. The proposed surgical alternative incorporates an additional graft passage between the tibia and fibula anteriorly (between the anterior aperture of the fibular tunnel and the anterior aperture of the tibial tunnel), aimed at stabilizing the PTFJ, which is thereby secured both anteriorly and posteriorly by the grafts. This technique employs two grafts, one reconstructing the LCL and the popliteofibular ligament/posterior proximal tibiofibular ligament, while the second reconstructs the popliteus tendon and the anterior proximal tibiofibular ligament. The following Figures 1 and 2 illustrate this biomechanical limitation and provide the rationale for a combined ligamentous reconstruction of the PLC and PTFJ.

Based on this conceptual framework, the proposed technique was performed in a fresh cadaveric model to illustrate the anatomic landmarks, graft configuration, tunnel placement, and fixation sequence required to achieve combined stabilization of the PLC and PTFJ.

The described surgical technique anatomically reconstructs the major stabilizing structures of the PLC and PTFJ, including the LCL, popliteus tendon, popliteofibular ligament, anterior proximal tibiofibular ligament, and posterior proximal tibiofibular ligament [2]. The reconstruction was performed using two tendon grafts, one to reconstruct the LCL and the PFL/PPTFL, and the second to reconstruct the PT and the APTFL (anterior proximal tibiofibular ligament). The PFL and PPTFJ (posterior proximal tibiofibular ligament) are considered a single functional structure in the reconstruction technique due to their similar anatomical course.

### 2.2 Specimen Preparation and Baseline Assessment

The procedure was performed on a male cadaveric specimen provided by the University of the Basque Country [Euskal Herriko Unibertsitatea (EHU)]. The left knee underwent reconstruction, while the contralateral limb served as the donor site for semitendinosus and gracilis tendon harvest [21]. Preoperative examination confirmed baseline stability of the PLC and PTFJ using standard clinical tests. This evaluation included the dial test at 30° and 90°, varus stress testing at 0°, 30°, 60°, and 90°, the posterolateral drawer test at 30° and 90°, the external rotation recurvatum test, and the PTFJ ballottement test with the knee flexed to 90°. These maneuvers confirmed intact stability before sectioning [22, 23, 24].

### 2.3 Graft Harvesting and Preparation

The specimen was positioned supine, with a lateral wedge to maintain internal rotation of the left knee. Tendon harvesting from the contralateral limb was performed using a standard technique with an Acufex tendon stripper (Smith & Nephew). Particular care was taken to maximize graft length, as each reconstruction required a minimum graft length of approximately 17 cm.

One graft was designed for the reconstruction of the LCL and PFL/PPTFL. The length requirement was calculated based on anatomical measurements: 20 mm required for the femoral fixation with an interference screw, 60 mm corresponding to the mean length of the LCL, 22.3 mm corresponding to the average anteroposterior diameter of the fibular head at its widest point (according to the location of the fibular tunnel), 11.8 mm corresponding to the mean length of the PFL (between the posterior aspect of the fibula and the posterior tibial surface where the musculotendinous junction is located), 48 mm corresponding to the average anteroposterior width of the lateral tibial plateau for the tibial tunnel, and 10 mm for screw fixation at the anterolateral tibia. Resulting in a total required graft length of approximately 17 cm [25, 26, 27, 28].

The second graft was used to reconstruct the PT and APTFL, and required a similar total length. This consisted of 20 mm for femoral fixation with an interference screw, 54.5 mm corresponding to the mean anatomical length of the popliteus tendon, 48 mm for the tibial tunnel length across the lateral tibial plateau, 30 mm from the anterior tibial tunnel exit to the anterior fibular tunnel entry, and 22.3 mm for the fibular tunnel length. Resulting in a total of approximately 17 cm [26, 28, 29].

Following harvest, both grafts were prepared with braided sutures using a whipstitch technique over the distal 3 cm of the graft ends. Graft diameters did not exceed 5 mm when measured with a sizing block. The grafts were subsequently mounted on an Acufex tensioning device and pre‐tensioned to approximately 70 N [30].

### 2.4 Surgical Approach and Structure Sectioning

A lateral hockey‐stick incision was performed, extending proximally along the iliotibial band, and distally between the fibular head and Gerdy's tubercle [31, 32]. Subcutaneous dissection was carried out to create a posteriorly based flap. The long head of the biceps femoris and the common peroneal nerve were identified, and neurolysis was performed. Blunt dissection allowed for the identification of the lateral gastrocnemius tendon, the posteromedial aspect of the fibular styloid, and the popliteus musculotendinous junction. Proximal dissection enabled identification and controlled sectioning of the LCL and PT. The capsule of the PTFJ was circumferentially released, effectively sectioning the APTFL, PPTFL, and the PFL. The femoral insertion sites of the LCL and PT, and the fibular insertion of the LCL, were preserved and marked to facilitate accurate tunnel placement. Following sectioning, instability of both the PLC and the PTFJ was confirmed through repetition of the previously described clinical tests.

### 2.5 Bone Tunnel Preparation

**FIGURE 3:**
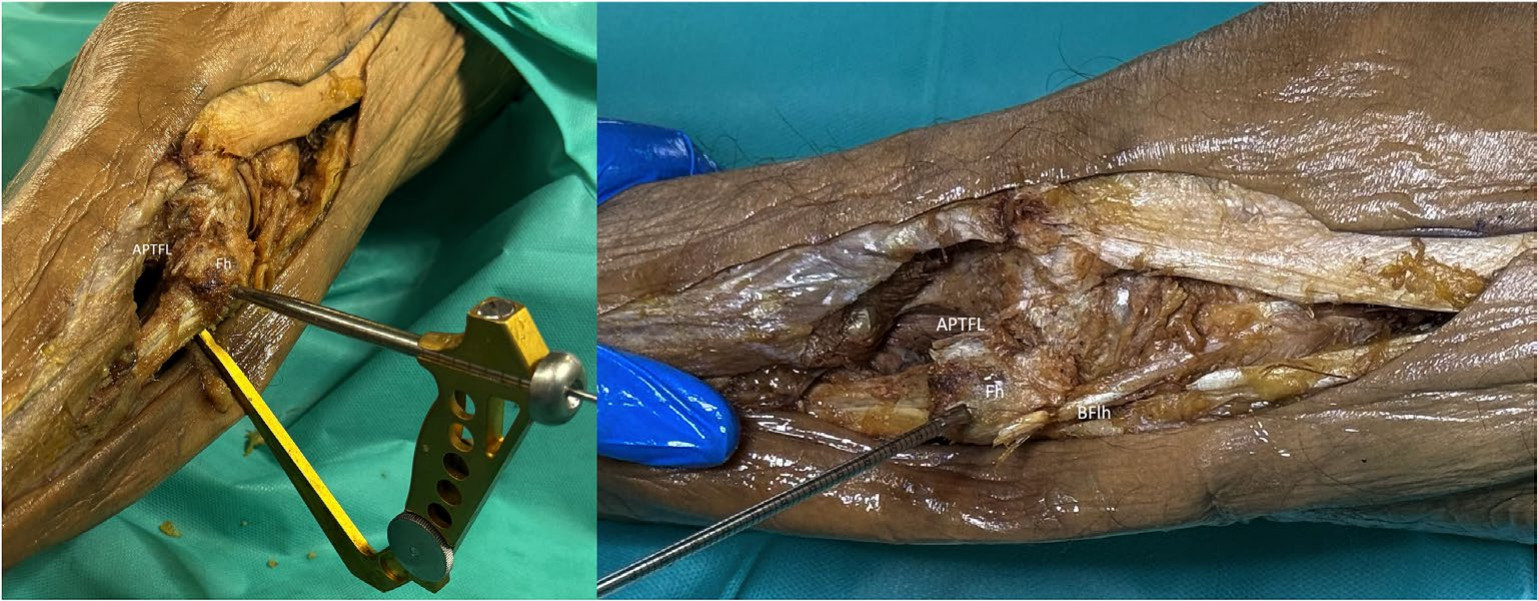
Left image: Preparation of the fibular tunnel. Placement of the guide pin from the anterolateral aspect of the fibula at the LCL insertion, directed posteromedially toward the fibular insertion of the popliteofibular ligament. Right image: Sequential reaming of the fibular head following the established trajectory. APTFL, anterior proximal tibiofibular ligament; BFlh, biceps femoris long head; Fh, fibular head.

Bone tunnel preparation was performed sequentially in the fibula, tibia, and femur. The fibular tunnel was drilled from the anterolateral to the posteromedial aspect of the fibular head (from the LCL insertion on the anterolateral fibular head to the PFL insertion on the posteromedial fibular head). An ACL aiming device (Acufex) was used to place a guide pin from the lateral aspect of the fibular head to the posteromedial downslope of the fibular styloid (Figure 3). The entry point was located immediately proximal to the “champagne glass drop‐off,” at the LCL attachment site, which is anatomically located 28.4 mm distal to the styloid tip and 8.2 mm posterior to the anterior margin of the fibular head. The tunnel was reamed to a diameter of 7 mm and progressively enlarged to 10 mm (Figure 3).

**FIGURE 4:**
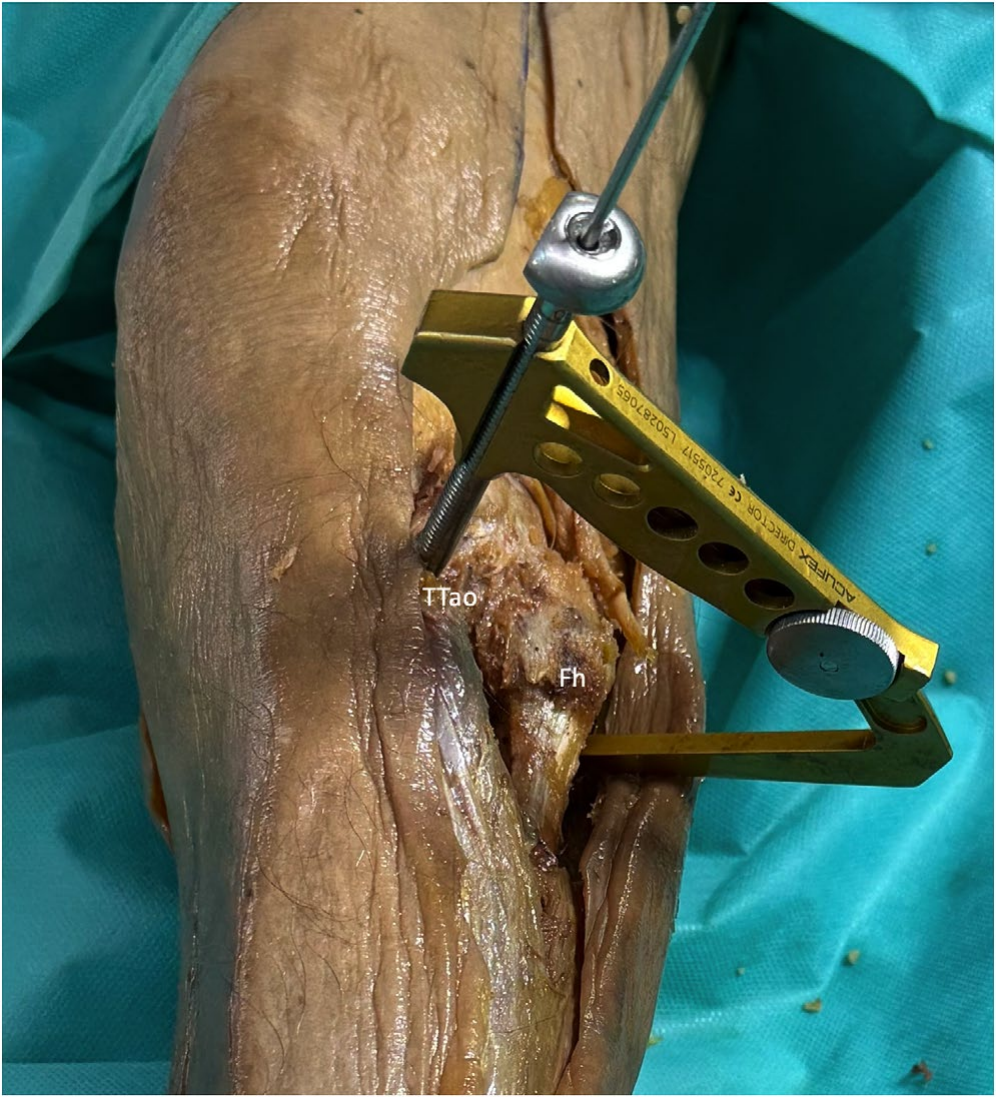
Tibial tunnel preparation, the orientation is established by placing a guide pin between Gerdy's tubercle and the anterior tibial tuberosity directed to the posterior tibial cortex at the popliteus musculotendinous junction, 1 cm proximal and 1 cm medial to the previously created fibular tunnel. Fh, fibular head; TTao, tibial tunnel anterior orifice.

The tibial tunnel was created next, the anterolateral tibial entry point was identified distal and medial to Gerdy's tubercle and just lateral to the tibial tubercle. The tunnel was directed toward a posterior exit point located 1 cm proximal and 1 cm medial to the fibular tunnel, corresponding to the musculotendinous junction of the popliteus. A guide pin was placed in an anterior‐to‐posterior direction using the ACL aiming device, and the tunnel was then progressively reamed to a diameter of 10 mm (Figure 4).

**FIGURE 5:**
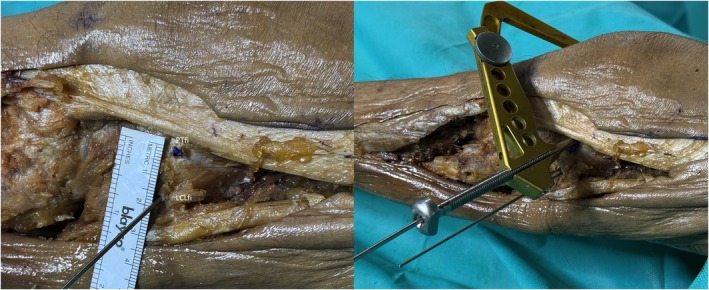
Left image: Placement of the femoral guide pin for the LCL tunnel, with a blue mark indicating the 18.5 mm anterior position of the popliteus tendon insertion in reference to the lateral collateral ligament insertion. Right image: Placement of the guide pin for the popliteus tendon femoral tunnel at its anatomic footprint. The tunnel is oriented parallel to the lateral collateral ligament tunnel. An anterior cruciate aiming guide is used to create both tunnels. LCLfi, lateral collateral ligament femoral insertion; PTfi, popliteus tendon femoral insertion.

Finally, femoral tunnels were created at the anatomical insertion sites of the LCL and PT. The LCL tunnel was drilled first using an ACL guide, directing the guide pin toward an exit point on the medial aspect of the distal femur, proximal and anterior to the entry point, in order to avoid convergence with the trochlea or a potential ACL tunnel. The femoral attachment of the PT was identified 18.5 mm anterior to the LCL insertion, within the anterior fifth of the popliteal sulcus, and a parallel guide pin was placed at this location. Both femoral tunnels were reamed to a diameter of 9 mm (Figure 5).

### 2.6 Graft Passage and Fixation Sequence

**FIGURE 6:**
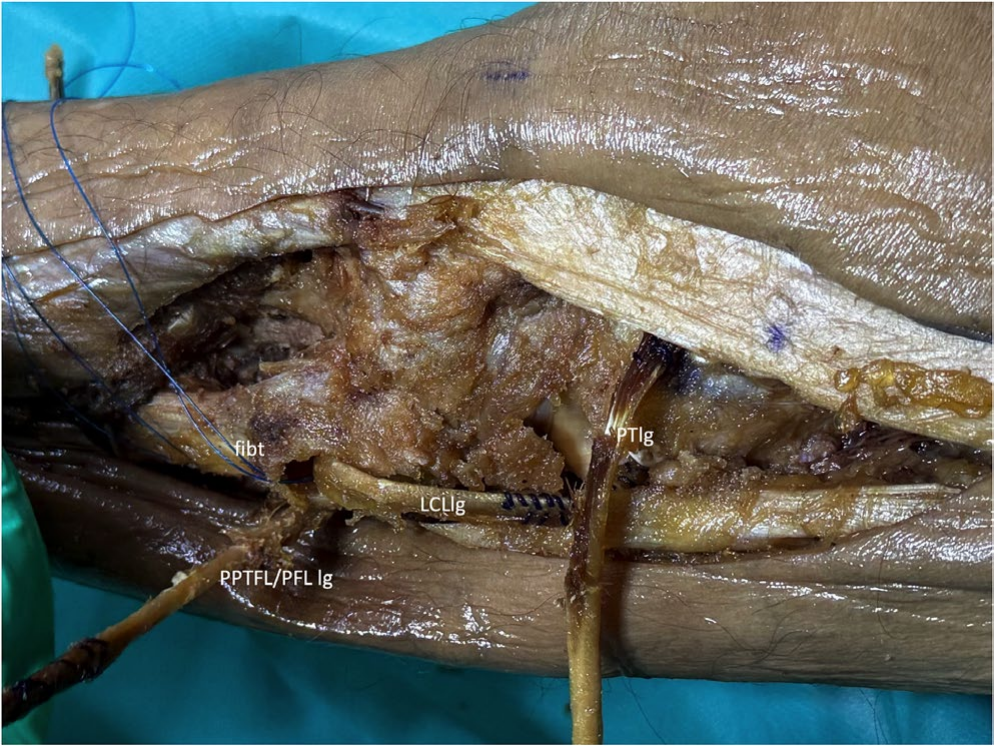
Ligamentous reconstruction process: The popliteus tendon graft is fixed at its anatomic femoral insertion. The LCL graft remains unfixed on its femoral insertion, but the opposite graft end is passed through the fibular head tunnel. Then, both grafts will traverse the tibial tunnel from the posterolateral tibial opening toward the anterolateral tibial opening. Fibt, fibular tunnel; LCLlg, lateral collateral ligament limb of the graft; PPTFJ/PFL lg, posterior proximal tibiofibular ligament/popliteofibular ligament limb of the graft; PTlg, popliteus tendon limb of the graft.

Both grafts were initially passed through their respective femoral tunnels and pulled medially. The PT graft was fixed first using a 10 × 30 mm interference screw, while the LCL graft was left unfixed at this stage, allowing for later tensioning (Figure 6).

The LCL–PFL/PPTFL graft was then passed through the fibular tunnel from anterior to posterior using a looped suture to pull the graft through the fibular tunnel. Subsequently, the PT–APTFL graft was passed deep to the LCL graft and directed toward the posterior tibial tunnel opening. Both grafts were passed simultaneously through the tibial tunnel from posterior to anterior, then it could be confirmed that the PT–APTFL graft remained longer than the LCL–PFL/PPTFL graft due to the different trajectories followed within the tunnels.

**FIGURE 7:**
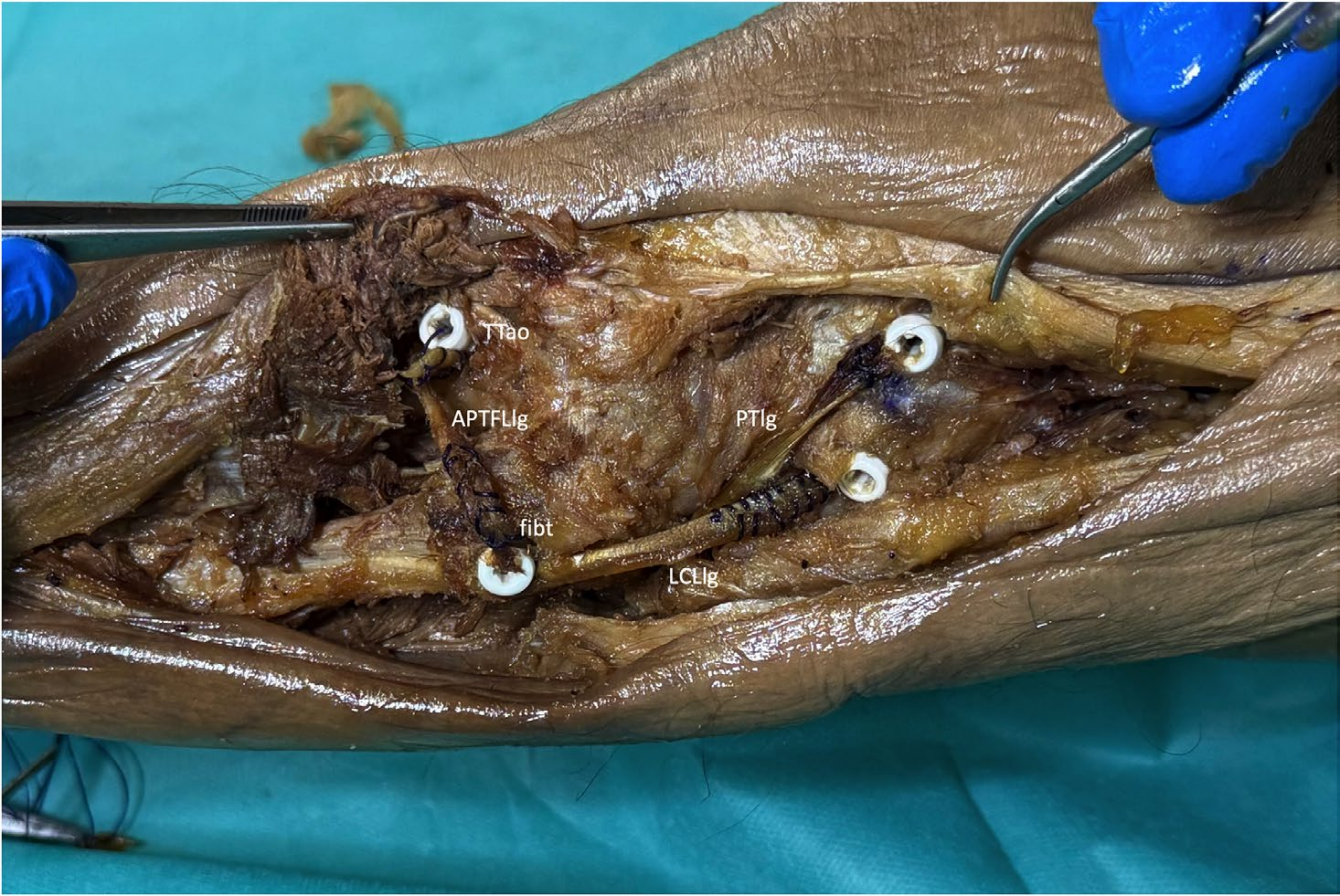
Complete anatomic reconstruction of the proximal tibiofibular joint and the PLC. The popliteus tendon graft, previously fixed at its femoral footprint, is routed toward the posterior tibial tunnel opening, crosses the tibial tunnel, and exits anterolaterally through the anterior tibial tunnel opening. At this point, the graft is secured at the fibular head, thereby reconstructing the anterior proximal tibiofibular ligament. Concurrently, the LCL graft originating from its femoral insertion traversed the fibular tunnel from anterior to posterior and crossed the tibial tunnel in a posterolateral‐to‐anterolateral direction before final fixation on the anterolateral tibia with an interference screw. The graft segment located between the posterior fibular tunnel opening and the posterior tibial tunnel opening reconstructs the posterior proximal tibiofibular ligament and popliteofibular ligament. APTFLgl, anterior proximal tibiofibular ligament limb of the graft; PTlg, popliteus tendon limb of the graft, fibt: fibular tunnel; TTao, tibial tunnel anterior orifice.

After several cycles of knee flexion and extension, both grafts were fixed at the anterolateral tibia using a single 10 × 30 mm interference screw with the knee flexed to 60°, securing the PT reconstruction. The remaining PT–APTFL graft was then passed through the fibular tunnel in an anterior‐to‐posterior direction using a looped suture to pull the graft through the tunnel. Following additional flexion–extension cycles, both grafts within the fibular tunnel were fixed using a 10 mm × 20 mm interference screw in an anterolateral‐to‐posteromedial direction with the knee flexed to 30°, completing fixation of the APTFL and PPTFL. Finally, after additional cycling, the LCL graft was tensioned and fixed in the femoral tunnel using a 10 mm × 30 mm interference screw with the knee at 30° of flexion, thereby completing the reconstruction (Figure 7).

### 2.7 Post‐Reconstruction Assessment

Post‐reconstruction, stability testing was assessed on the cadaveric knee specimen to evaluate the qualitative effectiveness of the combined surgical construct. Upon exploration, full restoration of joint stability and overall construct integrity was confirmed through the following specific outcome measures. Dial Test at 30° and 90°: Demonstrated a complete reduction of abnormal external rotation laxity compared to the unstable sectioned state, confirming restoration of the rotational restraints. Varus Stress Testing at 0°, 30°, 60°, and 90°: Confirmed stable endpoints and no joint opening throughout the range of motion. Posterolateral Drawer Test at 30° and 90°: Showed no residual abnormal posterior translation or external rotation during manual displacement, indicating stable PLC restraints. PTFJ Ballottement Test: Demonstrated the elimination of abnormal anteroposterior translation of the fibular head relative to the tibia, confirming stabilization of both the anterior and posterior proximal tibiofibular ligaments.

**FIGURE 8:**
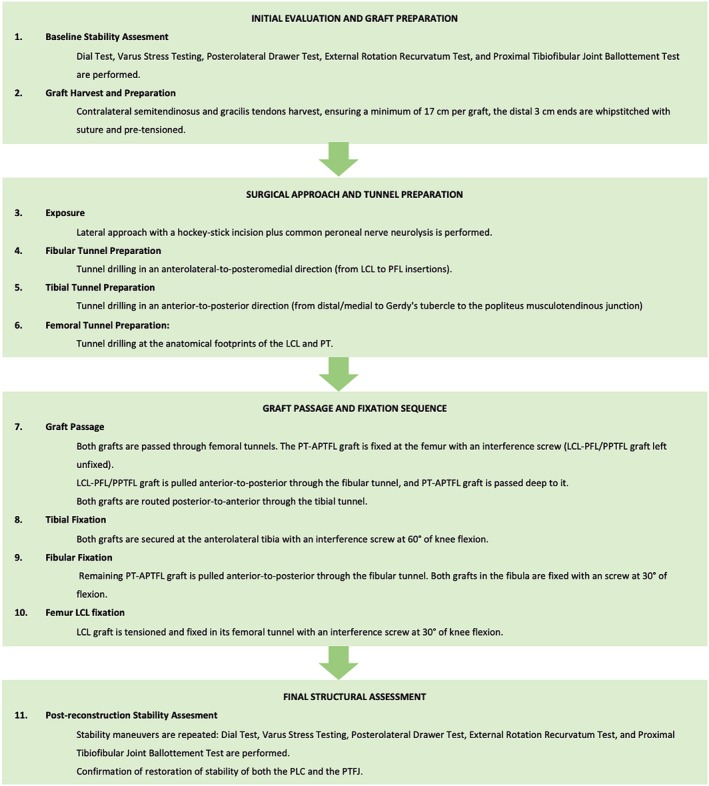
Schematic workflow of the combined reconstruction of the PTFJ and the posterolateral corner. APTFL, anterior proximal tibiofibular ligament; LCL, lateral collateral ligament; PFL, popliteofibular ligament; PLC, posterolateral corner; PPTFL, posterior proximal tibiofibular ligament; PT, popliteus tendon; PTFJ, proximal tibiofibular joint.

To facilitate a comprehensive understanding of the described technique, the surgical workflow is schematically illustrated in Figure 8.

## 3 Results

The proposed reconstruction described restored PLC and PTFJ manually tested qualitative stability in a cadaveric model, providing a potential surgical solution for a complex injury pattern for which no standardized treatment currently exists. The PLC reconstruction reproduced an anatomic tibiofibular‐based configuration, following the principles described by LaPrade et al. [3, 4, 5, 29, 31, 32, 33, 34, 35, 36]. Tunnel placement and graft passage were designed to restore the native anatomy of the LCL, popliteus tendon, and popliteofibular ligament, with the additional modification of the simultaneous reconstruction of the anterior and posterior proximal tibiofibular ligaments [25, 26, 31, 34]. This integrated approach allowed for the restoration of both the PLC and the PTFJ while maintaining an anatomic relationship between the fibula and tibia, which is critical given the functional interdependence of these structures.

A key feature of the described technique is the ability to independently tension and fix each reconstructed structure at different knee flexion angles, thereby optimizing graft tension according to the functional behavior of each component. The popliteus tendon was fixed at 60° of knee flexion, whereas the LCL and the anterior and posterior proximal tibiofibular ligaments were fixed at 30° of knee flexion. This angle‐specific fixation strategy reflects established biomechanical principles and represents a technical advantage of the technique.

The use of a two‐graft strategy provided sufficient graft length and facilitated independent tensioning of the reconstructed structures. This allowed each component to be fixed near its optimal functional position throughout the range of motion, which may contribute to improved biomechanical behavior compared with single‐graft or non‐anatomic constructs. Additional technical pearls and pitfalls for the technique are explained in Table 1.

**TABLE 1:** Technical pearls and pitfalls.

Pearls	Pitfalls
Marking of native femoral and fibular insertion sites before sectioning the PLC and PTFJ structures is recommended to facilitate accurate anatomic tunnel placement and reproducibility of the reconstruction	Malpositioned fibular tunnel may compromise both PLC and PTFJ reconstructions and significantly increase the risk of proximal fibular fracture, tunnel blowout, or non‐anatomic graft orientation, leading to residual instability or graft overload
Harvesting grafts with a minimum length of 17 cm is critical to allow passage through femoral, fibular, and tibial tunnels	Oversizing the fibular tunnel should be avoided. Passing two grafts through a single fibular tunnel requires careful control of graft diameter, as excessive tunnel enlargement weakens the fibular head. Graft diameters should be adapted to allow a tunnel size that preserves adequate bone stock
Pre‐placement of sutures attached to the grafts in the fibular and tibial tunnels before graft passage significantly simplifies the procedure by reducing graft manipulation and minimizing the risk of graft twisting and entanglement when grafts are passed through shared tunnels, also it allows controlled and sequential graft routing	
	Inadequate protection of the common peroneal nerve during posterior dissection and fibular tunnel preparation exposes the nerve to traction or direct injury
Independent tensioning and fixation of each reconstructed structure at specific knee flexion angles allows appropriate tension of the reconstructed structures while accommodating the physiological anterior–posterior translation of the fibular head during knee flexion. The popliteus tendon should be fixed at 60° of knee flexion, the fibular collateral ligament at 30°, and both anterior and posterior proximal tibiofibular ligaments at 30° as well	
	Insufficient graft length may force non‐anatomic fixation, excessive graft tension, or compromise the ability to independently tension each reconstructed structure
	Improper fixation angles can overconstrain the fibular head or alter normal kinematics either of the posterolateral corner or the proximal tibiofibular joint
Cycling the knee through a full range of motion before each fixation step allows graft creep to settle and avoids overconstraint of the reconstruction	

## 4 Discussion

The present study describes a novel, anatomically based surgical technique designed for the simultaneous reconstruction of the PTFJ and the PLC in the setting of combined knee instability. Utilizing a cadaveric model, this investigation demonstrated the technical feasibility of the combined procedure, which potentially restores knee stability and provides a standardized surgical solution for a complex injury pattern that currently lacks a consensus management protocol. Addressing PTFJ instability is considered paramount to maintaining the structural integrity and clinical efficacy of tibiofibular‐based PLC reconstructions, as persistent PTFJ laxity has been shown to compromise graft function and overall knee stability [2]. Structurally, the local synovial environment inherently limits the intrinsic healing capacity of the anterior and posterior tibiofibular ligaments, rendering spontaneous ligamentous healing or isolated primary repair highly unpredictable. To address these biological limitations, various PTFJ stabilization strategies have been proposed, each presenting distinct biomechanical implications that warrant careful consideration.

Regarding rigid screw fixation or proximal tibiofibular arthrodesis, this traditional approach induces a severe restriction on the physiological anteroposterior and rotational displacement of the fibular head during knee flexion and extension. Furthermore, it remains uncertain whether temporary rigid immobilization successfully facilitates capsular healing within a synovial environment, a question that requires deeper investigation. From a technical standpoint, rigid hardware exhibits a critically low tolerance for non‐anatomical reduction; any intraoperative malreduction is locked into place without a margin for physiological self‐adjustment, significantly escalating the risk of long‐term joint complications and hardware failure.

To mitigate the stiffness associated with rigid hardware, the implementation of a flexible suture‐button construct offers an alternative strategy with a superior biomechanical profile. By preserving a degree of physiological toggling and allowing the proximal fibula to pivot dynamically against the tibia, this dynamic system inherently accommodates minor intraoperative malreductions. Nevertheless, the underlying biological challenge persists, as flexible devices do not guarantee definitive capsular or ligamentous healing. In the event of a healing failure, long‐term PTFJ stability becomes entirely dependent on the suture material, leaving the mid‐to‐long‐term durability of the construct open to question.

In contrast to the aforementioned methods, the proposed anatomic ligamentous reconstruction emerges as the most promising therapeutic strategy. By optimizing graft positioning and tensioning, this technique effectively replicates and preserves the physiological kinematics of the PTFJ during knee motion, while demonstrating a favorable tolerance for minor malreductions due to its non‐rigid nature. Crucially, this approach directly addresses the biological limitations associated with poor ligamentous healing by physically replacing the damaged structures with a viable graft, the requirement for intrinsic biological healing is rendered obsolete, thereby mitigating a major mode of failure inherent to non‐reconstructive techniques.

The chosen fixation angles of the proposed PLC‐PTFJ reconstruction take into account the anteroposterior gliding motion of the proximal fibula during knee flexion and extension. With increasing knee flexion, the fibular head translates anteriorly due to the relaxation of the fibular collateral ligament and biceps femoris, whereas during extension it is displaced posteriorly as these structures become taut. Fixation of the posterior proximal tibiofibular ligament in a relatively flexed position allows for baseline tensioning in a biomechanically favorable position, as the fibular head is moving anteriorly at the time of fixation. This is particularly relevant given that several authors have identified the posterior proximal tibiofibular ligament as the primary stabilizer of the PTFJ, which is placed under tension with knee flexion [24, 37, 38]. The described sequence of fixation aims to restore physiological kinematics while minimizing the risk of overconstraint.

However, several limitations of the present study must be addressed. First, this investigation is restricted to a descriptive, single‐specimen cadaveric model and lacks quantitative biomechanical testing data. Joint stability was assessed exclusively via manual clinical testing, a method that is inherently subjective and potentially influenced by the specific tissue quality of the specimen. Consequently, these findings represent a qualitative evaluation rather than a precise biomechanical analysis.

Second, although the described technique permits variable fixation angles, the popliteofibular ligament graft was secured strictly at 30° of flexion. Given that previous literature indicates peak forces within the PFL occur at approximately 60°, fixation at higher degrees of flexion may be more biomechanically appropriate and warrants further investigation [39].

Third, the procedure requires the passage of two distinct grafts through a single 10‐mm fibular tunnel. While 5‐mm grafts were utilized in this model, larger graft diameters would necessitate wider tunnels, significantly increasing the risk of fibular head fracture or “tunnel blowout.” Even with the current dimensions, the risk of structural compromise to the fibula remains non‐negligible. To mitigate these risks, a preoperative computed tomography (CT) scan is highly advisable to evaluate the anatomy of the proximal fibula. This assessment must thoroughly quantify both the anteroposterior and mediolateral dimensions of the bone. In cases of diminished proximal fibular dimensions, or the presence of osteoporosis in this fibular region, this specific reconstruction technique should be discouraged to prevent intraoperative proximal fractures that would ultimately compromise the surgical objective.

Furthermore, strict intraoperative verification of the safe zone for the common peroneal nerve is mandatory. A comprehensive understanding of its precise location and anatomical trajectory is critical to avoid iatrogenic injury during tunnel creation and graft manipulation, thereby requiring continuous nerve protection throughout the entire procedure.

Lastly, securing backup graft options prior to surgery is highly relevant. The substantial graft demand of this procedure presents a major limitation, particularly in multiligamentous knee injuries where concurrent anterior cruciate ligament or posterior cruciate ligament reconstructions are required. Consequently, preoperative planning must anticipate the potential need for allografts of sufficient length or, alternatively, the harvesting of long autografts, such as the plantaris or peroneus longus tendons [40, 41, 42].

Additionally, this technique has not been validated clinically in patients or in vivo; therefore, its clinical feasibility, long‐term durability, complication rates, and functional outcomes remain unknown. Despite its inherent limitations, the proposed technique establishes a reproducible, anatomically based framework for addressing this complex injury pattern, thereby serving as a foundational basis for future research. Given that the present study represents a proof‐of‐concept investigation, further rigorous biomechanical validation, alongside in vivo models and clinical studies, remains imperative before its clinical implementation can be recommended.

In conclusion, this study describes an anatomic reconstruction technique for the simultaneous treatment of PLC and PTFJ instability, providing a potential surgical approach for this complex injury pattern. The proposed technique preserves physiological PTFJ mobility while establishing a stable fibular‐based construct for PLC reconstruction. A distinctive feature of the procedure is the angle‐specific fixation strategy, which enables independent graft tensioning according to the functional behavior of the reconstructed structures and respects the native anteroposterior kinematics of the fibular head.

However, the findings of this study must be interpreted in light of its principal limitation: the technique was developed and evaluated in a single cadaveric specimen. Consequently, no conclusions can be drawn regarding reproducibility, biomechanical performance, or clinical effectiveness. Furthermore, several technical challenges may arise in clinical practice, including the risk of fibular tunnel convergence or compromise and the substantial graft length required for reconstruction.

Despite these limitations, the successful execution of the procedure in a cadaveric setting demonstrates its technical feasibility and provides an anatomic framework for the management of combined PLC and PTFJ instability. Further biomechanical investigations and clinical studies are necessary to assess the reproducibility, durability, and functional outcomes of this reconstruction strategy before its clinical adoption can be recommended.

## Author Contributions

**Javier Faus‐Cotino:** conceptualization, investigation, funding acquisition, writing – original draft, methodology, validation, visualization, writing – review and editing. **Ainhoa Amarilla‐Irusta:** conceptualization, investigation, writing – review and editing. **Isabel Andia:** conceptualization, validation, writing – review and editing. **José Antonio Guerrero Serrano:** conceptualization, writing – review and editing. **Aingeru Sarriugarte Lasarte:** writing – review and editing, conceptualization.

## Funding

This research received no external funding. For the performance of the procedure, surgical instrumentation and fixation materials were supplied by Smith & Nephew.

## Disclosure

All authors listed meet the authorship criteria according to the latest guidelines of the International Committee of Medical Journal Editors, and all authors are in agreement with the manuscript.

## Conflicts of Interest

The authors declare no conflicts of interest.

## Data Availability

The data that support the findings of this study are openly available in Pubmed at https://pubmed.ncbi.nlm.nih.gov/.
